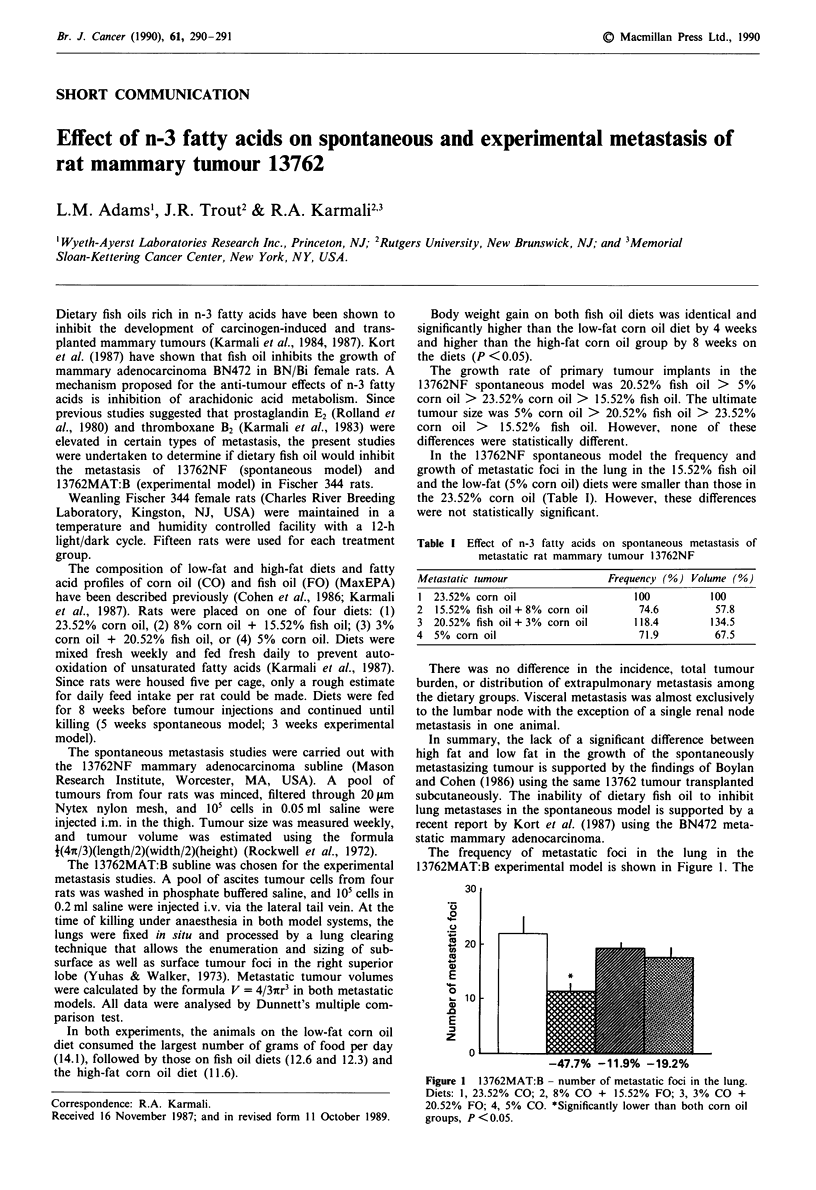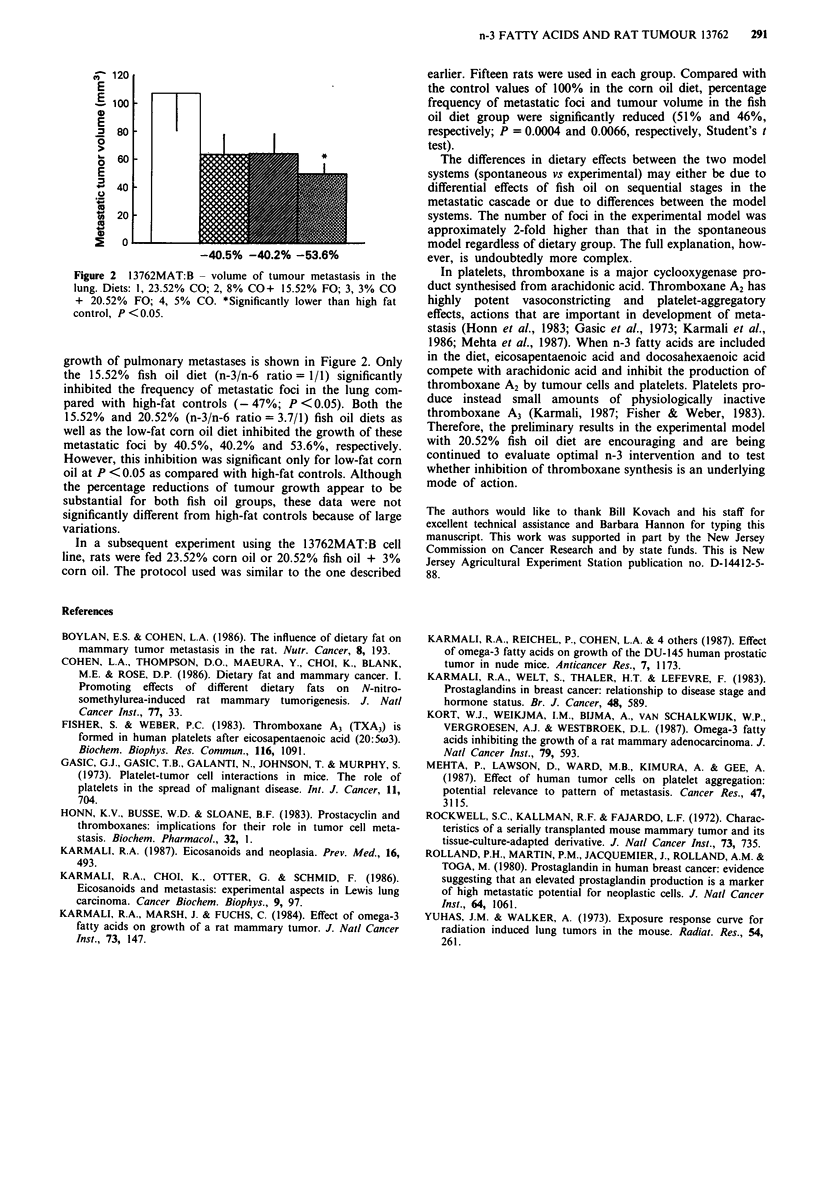# Effect of n-3 fatty acids on spontaneous and experimental metastasis of rat mammary tumour 13762.

**DOI:** 10.1038/bjc.1990.54

**Published:** 1990-02

**Authors:** L. M. Adams, J. R. Trout, R. A. Karmali

**Affiliations:** Wyeth-Ayerst Laboratories Research Inc., Princeton, NJ.


Br. J. Cancer (1990), 61, 290-291 © Macmillan Press Ltd., 1990

SHORT COMMUNICATION

### Effect of n-3 fatty acids on spontaneous and experimental metastasis of rat mammary tumour 13762

L.M. Adams[1], J.R. Trout[2] & R.A. Karmali[2,3]

[1]*Wyeth-Ayerst Laboratories Research Inc., Princeton, NJ;* [2]*Rutgers University, New Brunswick, NJ; and* [3]*Memorial Sloan-Kettering Cancer Center, New York, NY, USA.*

Dietary fish oils rich in n-3 fatty acids have been shown to inhibit the development of carcinogen-induced and transplanted mammary tumours (Karmali *et al.*, 1984, 1987). Kort *et al.* (1987) have shown that fish oil inhibits the growth of mammary adenocarcinoma BN472 in BN/Bi female rats. A mechanism proposed for the anti-tumour effects of n-3 fatty acids is inhibition of arachidonic acid metabolism. Since previous studies suggested that prostaglandin $E_2$ (Rolland *et al.*, 1980) and thromboxane $B_2$ (Karmali *et al.*, 1983) were elevated in certain types of metastasis, the present studies were undertaken to determine if dietary fish oil would inhibit the metastasis of 13762NF (spontaneous model) and 13762MAT:B (experimental model) in Fischer 344 rats.

Weanling Fischer 344 female rats (Charles River Breeding Laboratory, Kingston, NJ, USA) were maintained in a temperature and humidity controlled facility with a 12-h light/dark cycle. Fifteen rats were used for each treatment group.

The composition of low-fat and high-fat diets and fatty acid profiles of corn oil (CO) and fish oil (FO) (MaxEPA) have been described previously (Cohen *et al.*, 1986; Karmali *et al.*, 1987). Rats were placed on one of four diets: (1) 23.52% corn oil, (2) 8% corn oil + 15.52% fish oil; (3) 3% corn oil + 20.52% fish oil, or (4) 5% corn oil. Diets were mixed fresh weekly and fed fresh daily to prevent auto-oxidation of unsaturated fatty acids (Karmali *et al.*, 1987). Since rats were housed five per cage, only a rough estimate for daily feed intake per rat could be made. Diets were fed for 8 weeks before tumour injections and continued until killing (5 weeks spontaneous model; 3 weeks experimental model).

The spontaneous metastasis studies were carried out with the 13762NF mammary adenocarcinoma subline (Mason Research Institute, Worcester, MA, USA). A pool of tumours from four rats was minced, filtered through 20 μm Nytex nylon mesh, and $10^5$ cells in 0.05 ml saline were injected i.m. in the thigh. Tumour size was measured weekly, and tumour volume was estimated using the formula $\frac{1}{2}(4\pi/3)$(length/2)(width/2)(height) (Rockwell *et al.*, 1972).

The 13762MAT:B subline was chosen for the experimental metastasis studies. A pool of ascites tumour cells from four rats was washed in phosphate buffered saline, and $10^5$ cells in 0.2 ml saline were injected i.v. via the lateral tail vein. At the time of killing under anaesthesia in both model systems, the lungs were fixed *in situ* and processed by a lung clearing technique that allows the enumeration and sizing of sub-surface as well as surface tumour foci in the right superior lobe (Yuhas & Walker, 1973). Metastatic tumour volumes were calculated by the formula $V = 4/3\pi r^3$ in both metastatic models. All data were analysed by Dunnett's multiple comparison test.

In both experiments, the animals on the low-fat corn oil diet consumed the largest number of grams of food per day (14.1), followed by those on fish oil diets (12.6 and 12.3) and the high-fat corn oil diet (11.6).

Correspondence: R.A. Karmali.
Received 16 November 1987; and in revised form 11 October 1989.

Body weight gain on both fish oil diets was identical and significantly higher than the low-fat corn oil diet by 4 weeks and higher than the high-fat corn oil group by 8 weeks on the diets ($P < 0.05$).

The growth rate of primary tumour implants in the 13762NF spontaneous model was 20.52% fish oil > 5% corn oil > 23.52% corn oil > 15.52% fish oil. The ultimate tumour size was 5% corn oil > 20.52% fish oil > 23.52% corn oil > 15.52% fish oil. However, none of these differences were statistically different.

In the 13762NF spontaneous model the frequency and growth of metastatic foci in the lung in the 15.52% fish oil and the low-fat (5% corn oil) diets were smaller than those in the 23.52% corn oil (Table I). However, these differences were not statistically significant.

**Table I** Effect of n-3 fatty acids on spontaneous metastasis of metastatic rat mammary tumour 13762NF

| *Metastatic tumour* | *Frequency (%)* | *Volume (%)* |
|---|---|---|
| 1 23.52% corn oil | 100 | 100 |
| 2 15.52% fish oil + 8% corn oil | 74.6 | 57.8 |
| 3 20.52% fish oil + 3% corn oil | 118.4 | 134.5 |
| 4 5% corn oil | 71.9 | 67.5 |

There was no difference in the incidence, total tumour burden, or distribution of extrapulmonary metastasis among the dietary groups. Visceral metastasis was almost exclusively to the lumbar node with the exception of a single renal node metastasis in one animal.

In summary, the lack of a significant difference between high fat and low fat in the growth of the spontaneously metastasizing tumour is supported by the findings of Boylan and Cohen (1986) using the same 13762 tumour transplanted subcutaneously. The inability of dietary fish oil to inhibit lung metastases in the spontaneous model is supported by a recent report by Kort *et al.* (1987) using the BN472 metastatic mammary adenocarcinoma.

The frequency of metastatic foci in the lung in the 13762MAT:B experimental model is shown in Figure 1. The

**Figure 1** 13762MAT:B – number of metastatic foci in the lung. Diets: 1, 23.52% CO; 2, 8% CO + 15.52% FO; 3, 3% CO + 20.52% FO; 4, 5% CO. *Significantly lower than both corn oil groups, $P < 0.05$.

**Figure 2** 13762MAT:B – volume of tumour metastasis in the lung. Diets: 1, 23.52% CO; 2, 8% CO+ 15.52% FO; 3, 3% CO + 20.52% FO; 4, 5% CO. *Significantly lower than high fat control, $P < 0.05$.

growth of pulmonary metastases is shown in Figure 2. Only the 15.52% fish oil diet (n-3/n-6 ratio = 1/1) significantly inhibited the frequency of metastatic foci in the lung compared with high-fat controls (− 47%; $P < 0.05$). Both the 15.52% and 20.52% (n-3/n-6 ratio = 3.7/1) fish oil diets as well as the low-fat corn oil diet inhibited the growth of these metastatic foci by 40.5%, 40.2% and 53.6%, respectively. However, this inhibition was significant only for low-fat corn oil at $P < 0.05$ as compared with high-fat controls. Although the percentage reductions of tumour growth appear to be substantial for both fish oil groups, these data were not significantly different from high-fat controls because of large variations.

In a subsequent experiment using the 13762MAT:B cell line, rats were fed 23.52% corn oil or 20.52% fish oil + 3% corn oil. The protocol used was similar to the one described earlier. Fifteen rats were used in each group. Compared with the control values of 100% in the corn oil diet, percentage frequency of metastatic foci and tumour volume in the fish oil diet group were significantly reduced (51% and 46%, respectively; $P = 0.0004$ and 0.0066, respectively, Student's *t* test).

The differences in dietary effects between the two model systems (spontaneous *vs* experimental) may either be due to differential effects of fish oil on sequential stages in the metastatic cascade or due to differences between the model systems. The number of foci in the experimental model was approximately 2-fold higher than that in the spontaneous model regardless of dietary group. The full explanation, however, is undoubtedly more complex.

In platelets, thromboxane is a major cyclooxygenase product synthesised from arachidonic acid. Thromboxane $A_2$ has highly potent vasoconstricting and platelet-aggregatory effects, actions that are important in development of metastasis (Honn *et al.*, 1983; Gasic *et al.*, 1973; Karmali *et al.*, 1986; Mehta *et al.*, 1987). When n-3 fatty acids are included in the diet, eicosapentaenoic acid and docosahexaenoic acid compete with arachidonic acid and inhibit the production of thromboxane $A_2$ by tumour cells and platelets. Platelets produce instead small amounts of physiologically inactive thromboxane $A_3$ (Karmali, 1987; Fisher & Weber, 1983). Therefore, the preliminary results in the experimental model with 20.52% fish oil diet are encouraging and are being continued to evaluate optimal n-3 intervention and to test whether inhibition of thromboxane synthesis is an underlying mode of action.

The authors would like to thank Bill Kovach and his staff for excellent technical assistance and Barbara Hannon for typing this manuscript. This work was supported in part by the New Jersey Commission on Cancer Research and by state funds. This is New Jersey Agricultural Experiment Station publication no. D-14412-5-88.

### References

BOYLAN, E.S. & COHEN, L.A. (1986). The influence of dietary fat on mammary tumor metastasis in the rat. *Nutr. Cancer*, **8**, 193.

COHEN, L.A., THOMPSON, D.O., MAEURA, Y., CHOI, K., BLANK, M.E. & ROSE, D.P. (1986). Dietary fat and mammary cancer. I. Promoting effects of different dietary fats on *N*-nitrosomethylurea-induced rat mammary tumorigenesis. *J. Natl Cancer Inst.*, **77**, 33.

FISHER, S. & WEBER, P.C. (1983). Thromboxane $A_3$ ($TXA_3$) is formed in human platelets after eicosapentaenoic acid (20:5ω3). *Biochem. Biophys. Res. Commun.*, **116**, 1091.

GASIC, G.J., GASIC, T.B., GALANTI, N., JOHNSON, T. & MURPHY, S. (1973). Platelet-tumor cell interactions in mice. The role of platelets in the spread of malignant disease. *Int. J. Cancer*, **11**, 704.

HONN, K.V., BUSSE, W.D. & SLOANE, B.F. (1983). Prostacyclin and thromboxanes: implications for their role in tumor cell metastasis. *Biochem. Pharmacol.*, **32**, 1.

KARMALI, R.A. (1987). Eicosanoids and neoplasia. *Prev. Med.*, **16**, 493.

KARMALI, R.A., CHOI, K., OTTER, G. & SCHMID, F. (1986). Eicosanoids and metastasis: experimental aspects in Lewis lung carcinoma. *Cancer Biochem. Biophys.*, **9**, 97.

KARMALI, R.A., MARSH, J. & FUCHS, C. (1984). Effect of omega-3 fatty acids on growth of a rat mammary tumor. *J. Natl Cancer Inst.*, **73**, 147.

KARMALI, R.A., REICHEL, P., COHEN, L.A. & 4 others (1987). Effect of omega-3 fatty acids on growth of the DU-145 human prostatic tumor in nude mice. *Anticancer Res.*, **7**, 1173.

KARMALI, R.A., WELT, S., THALER, H.T. & LEFEVRE, F. (1983). Prostaglandins in breast cancer: relationship to disease stage and hormone status. *Br. J. Cancer*, **48**, 589.

KORT, W.J., WEIKJMA, I.M., BIJMA, A., VAN SCHALKWIJK, W.P., VERGROESEN, A.J. & WESTBROEK, D.L. (1987). Omega-3 fatty acids inhibiting the growth of a rat mammary adenocarcinoma. *J. Natl Cancer Inst.*, **79**, 593.

MEHTA, P., LAWSON, D., WARD, M.B., KIMURA, A. & GEE, A. (1987). Effect of human tumor cells on platelet aggregation: potential relevance to pattern of metastasis. *Cancer Res.*, **47**, 3115.

ROCKWELL, S.C., KALLMAN, R.F. & FAJARDO, L.F. (1972). Characteristics of a serially transplanted mouse mammary tumor and its tissue-culture-adapted derivative. *J. Natl Cancer Inst.*, **73**, 735.

ROLLAND, P.H., MARTIN, P.M., JACQUEMIER, J., ROLLAND, A.M. & TOGA, M. (1980). Prostaglandin in human breast cancer: evidence suggesting that an elevated prostaglandin production is a marker of high metastatic potential for neoplastic cells. *J. Natl Cancer Inst.*, **64**, 1061.

YUHAS, J.M. & WALKER, A. (1973). Exposure response curve for radiation induced lung tumors in the mouse. *Radiat. Res.*, **54**, 261.